# Source Apportionment of Heavy Metals in Stream Sediments from the Hilly Western Wanda Mountains Based on PCA-APCS-MLR

**DOI:** 10.3390/toxics14080696

**Published:** 2026-08-06

**Authors:** Xiangjin Yan, Lian Xue, Qi Wang, Jinyong Wang, Jingtao Shi, Chuangchuang Liu

**Affiliations:** 1 Mudanjiang Natural Resources Comprehensive Survey Center, China Geological Survey, Changchun 130102, China; yanx9203@163.com; 2 Water Conservancy Bureau of Hulunbuir City, Hulunbuir 021000, China; xuelian2013111@163.com; 3 Hulunbuir Water Conservancy Development Center, Hulunbuir 021000, China; 13947072427@163.com; 4 Langfang Natural Resources Comprehensive Survey Center, China Geological Survey, Tianjin 300301, China; 19134123625@189.cn

**Keywords:** stream sediments, heavy metals, PCA-APCS-MLR, source apportionment, ecological risk, Western foothills of the Wanda Mountains

## Abstract

The hilly terrain at the western foot of the Wanda Mountains boasts intricate geological conditions, compounded by long-term mineral extraction and farming activities. The multi-sourced heavy metals stored within stream sediments create substantial obstacles to accurate source apportionment and targeted risk mitigation. In this work, stream sediment samples were collected across the study watershed to test concentrations of eight heavy metals: As, Cd, Cr, Ni, Cu, Pb, Hg and Zn. Pollution and ecological risk assessments were implemented via the pollution load index (*PLI*), geo-accumulation index (*I_geo_*) and potential ecological risk index (*RI*). Kriging interpolation was utilized to visualize and interpret the spatial variation patterns of heavy metals. Combined correlation analysis, principal component analysis (PCA) and the Absolute Principal Component Scores–Multiple Linear Regression (APCS-MLR) receptor model were adopted to qualitatively and quantitatively distinguish pollution endmembers of heavy metals. The main results are as follows: Cr, Cd, Pb and As show significant enrichment, whereas Ni, Cu, Zn and Hg appear depleted; all measured heavy metals present strong spatial variability. Weathering of basic bedrocks governs the widespread high-value zones of Cr, Ni, Cu and Zn. Patchy enrichment of Pb, As and Cd is concentrated in river valley farmlands, with no continuous anomalous zones detected for Hg. The watershed as a whole suffers mild contamination and low potential ecological risk, with only sampling points surrounding mines and agricultural lands experiencing moderate-to-severe pollution and medium ecological hazards. Three source endmembers are identified in the region: a natural geogenic endmember (Cr, Ni, Co, etc.), a mixed mining-associated anthropogenic endmember (Zn, Hg, Cu, Cd), and an agricultural non-point endmember (Pb, As, Sb). Quantitative APCS-MLR calculations confirm that mining disturbances serve as the predominant anthropogenic input for Zn, Hg, Cu and Cd; agricultural practices dominate the accumulation of As and Pb; and geogenic contributions stay marginal for all elements. This study corroborates that regional lithology sets the natural geochemical baseline of heavy metals, while anthropogenic interferences function as the primary driver of localized pollution anomalies. The conclusions can deliver theoretical support for heavy metal pollution remediation in similar hilly watersheds throughout Northeast China.

## 1. Introduction

As an integral component of watershed ecosystems, stream sediments serve as both major sinks and potential sources of heavy metals, preserving long-term records of regional heavy metal enrichment, migration behaviors and pollution evolutionary trends [1,2]. Their heavy metal pollution status is directly related to watershed water environmental safety, the stability of aquatic biotic communities, and regional residential ecological health [3]. Heavy metals feature high toxicity, recalcitrance, environmental persistence, strong bioaccumulation capacity, and trophic biomagnification across food webs. Excessive accumulation of these metals in water bodies and stream sediments would pose severe hazards to aquatic ecosystems and human health [4,5,6]. Changes in aquatic environmental conditions (e.g., pH, redox potential, and organic matter decomposition) may remobilize immobilized heavy metals from stream sediments into the overlying water column, inducing secondary pollution and destabilizing aquatic ecological balance [7,8].

Currently, with the continuous advancement of regional industrialization, urbanization, mineral exploitation and modern agricultural activities, anthropogenic inputs have become the dominant driver of heavy metal pollution in watersheds [9,10]. Especially in hilly mountainous regions with complex geological settings where human activities overlap with natural processes, heavy metal sources exhibit diversified and complicated characteristics [11,12]. Therefore, accurately identifying pollution sources and quantifying the contribution ratios of various source categories constitute a crucial step for watershed heavy metal pollution remediation [13]. Failure to distinguish the respective contributions of natural geochemical background and anthropogenic contamination may readily lead to misjudgments such as attributing high geological background values to human pollution and ignoring hidden anthropogenic sources, thereby undermining the scientific rationality and effectiveness of pollution control strategies [14].

Numerous scholars have conducted extensive research on heavy metal contamination in various types of stream sediments, including fluvial, lacustrine and coastal sediments, and established a set of mature pollution and risk assessment systems such as the geo-accumulation index (*Igeo*), pollution load index (*PLI*) and potential ecological risk index (*RI*) [15,16,17]. These assessment methods can quantify the hazard levels of heavy metals in stream sediments from multiple dimensions, including single-element pollution degree, comprehensive pollution load and biotoxic risk, and provide fundamental data support for regional pollution management and control [18,19]. Nevertheless, traditional pollution assessment methods can only qualitatively characterize the current pollution status and fail to effectively differentiate geogenic and anthropogenic sources, making precise control over pollution sources unattainable [20]. Within the technical framework for heavy metal source apportionment, multivariate statistical models are widely adopted in source identification studies of environmental media such as soils and stream sediments, owing to their merits including easy operation, high analytical accuracy and suitability for large sample datasets [21,22]. Pearson correlation analysis and principal component analysis (PCA) serve as classic approaches to identify elemental paragenetic combinations and screen potential pollution sources. Relying on elemental geochemical correlations, they enable qualitative differentiation of geogenic, industrial, agricultural and other source categories [23,24]. By contrast, the APCS-MLR model improves upon conventional PCA by removing background interference, quantifying source contributions to each heavy metal, and bridging qualitative source discrimination and quantitative apportionment [25,26]. Owing to strong stability and superior precision, this integrated method is extensively utilized to trace heavy metal origins in watersheds, soils and sediments, emerging as the mainstream combined model for heavy metal source apportionment of stream sediments [27,28].

Mountains, hills and plains interlace across Northeast China, where dense drainage networks prevail. Intensive weathering and fluvial transport occur under a cold temperate monsoon climate. Combined with rich mineral reserves and large-scale farming, watershed stream sediments suffer from complicated environmental problems driven by overlapping geogenic high background and human-induced pollution [29]. Located at the transition from low hills to the Sanjiang Plain, the western piedmont hilly zone of the Wanda Mountains has flat terrain and well-developed gullies and rivers. Widespread farmlands, forests and mineral deposits coexist here, where coupled geogenic backgrounds and human activities facilitate heavy metal migration and accumulation [30]. Diverse strata are exposed in this area, including Proterozoic metamorphics, Cretaceous volcanics, Neogene basalts and multi-phase intrusions. Variable heavy metal geogenic backgrounds stem from complicated lithological combinations; weathering of distinct parent rocks releases divergent metallic elements and generates regional differentiated high geochemical baselines [31]. Meanwhile, sustained farming and mineral exploitation in this area introduce exogenous heavy metals through agrochemical application, mine wastewater discharge, tailing weathering–leaching and surface fluvial transport. Heavy metals in stream sediments are thus controlled by coupled geogenic and anthropogenic processes, significantly complicating source identification and pollution risk management [32].

This paper takes stream sediments from the hilly region on the western foot of the Wanda Mountains as the research object and determines heavy metal concentrations based on field-measured data. Combined with Kriging interpolation, multiple pollution and ecological risk assessment methods are adopted to analyze the pollution status, ecological risks and spatial distribution characteristics of heavy metals. Pearson correlation analysis, principal component analysis and the APCS-MLR model are applied to qualitatively and quantitatively identify heavy metal pollution sources and their respective source contributions. This study clarifies the dominant factors controlling regional heavy metal enrichment, distinguishes the impacts of geogenic background and anthropogenic contamination, and proposes differentiated prevention, control and remediation strategies. The research results can provide theoretical support and technical references for watershed environmental management, local resource exploitation, and heavy metal pollution control in analogous regions of Northeast China.

## 2. Study Area Setting

The study area is located in the transition zone between the western piedmont hills of the Wanda Mountains and the Sanjiang Plain, covering an area of 350 km^2^. The terrain generally slopes from northeast to southwest with elevations ranging from 150 m to 450 m. Low hills and rolling plains constitute the main landforms, with well-developed gullies and terraces and gentle topographic relief. The region features a cold temperate continental monsoon climate with four distinct seasons. The annual average temperature is 3.9 °C and the annual precipitation is 550 mm. Rainfall is concentrated in summer, forming a simultaneous period of abundant rainfall and high temperature. The dominant soil types within the study area include black soil, albic soil and meadow soil. These soils have deep layers and high fertility, making them suitable for agricultural production. Large contiguous farmlands are distributed across the region, where corn, soybean and other crops are mainly cultivated. In addition, forest and grassland resources are abundant.

**Figure 1:**
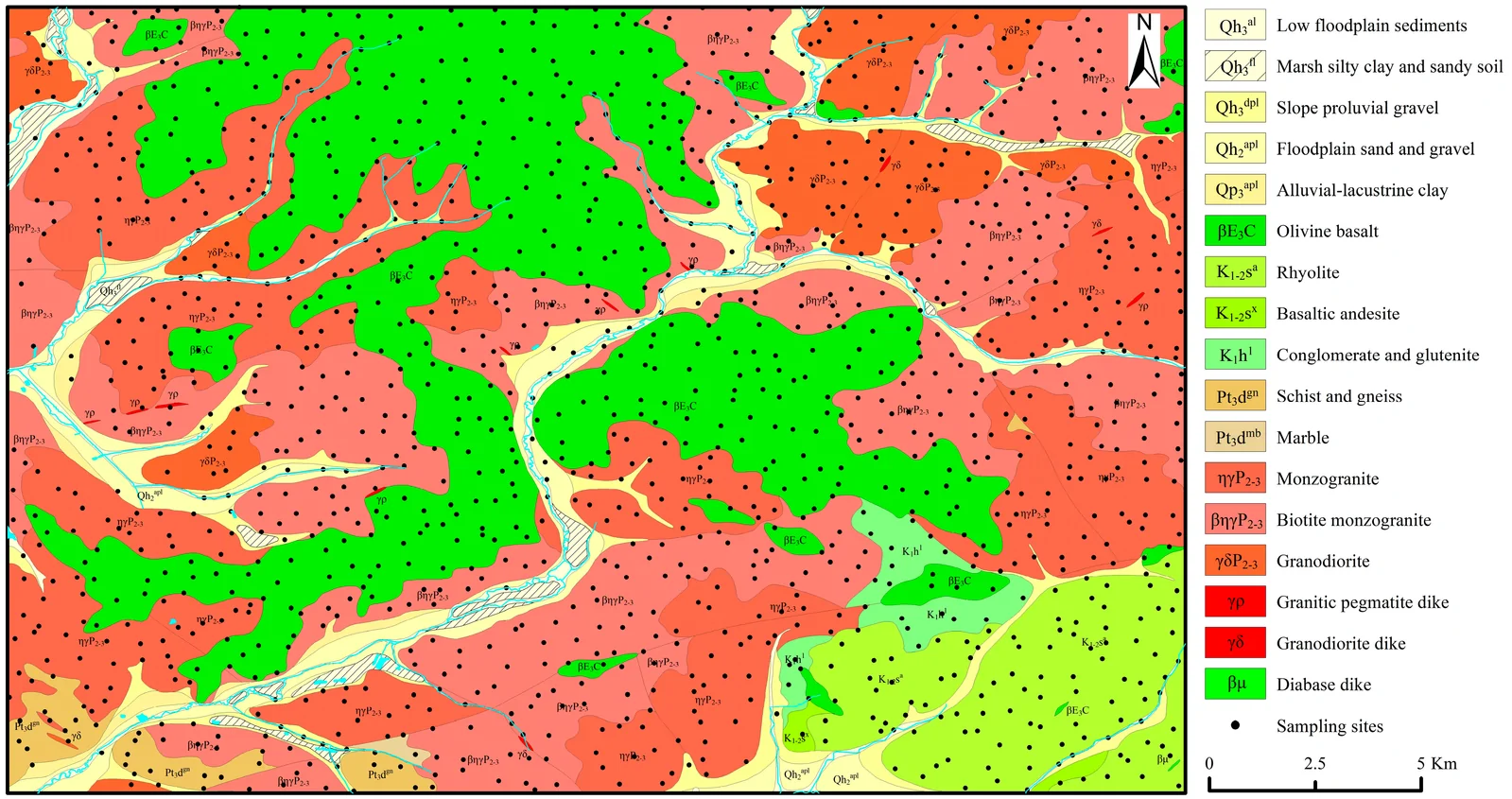
Simplified geological map and sampling locations of the study area.

The study area is situated within the Mengjiagang-Qixingpao magmatic arc tectonic unit, featuring a wide span of stratigraphic ages with outcrops of Proterozoic, Cretaceous, Neogene and Quaternary strata. The Proterozoic Dapandao Formation consists of metamorphic rocks. The Cretaceous sequence is dominated by continental volcanic rocks and fluvio-lacustrine clastic rocks. Basalts of the Neogene Chuandishan Formation are the most widely distributed lithology, while the Quaternary is composed of modern unconsolidated sediments. Intrusive rocks in the area are predominantly Middle–Late Permian crustal deep-seated granites (Figure 1).

## 3. Materials and Methods

### 3.1. Distribution of Sampling Sites and Sample Collection

#### 3.1.1. Sampling Grid and Site Layout

Stream sediment sampling was carried out in strict accordance with the Specifications for Regional Geochemical Survey at the Scale of 1:50,000 (DZ/T 0011-2015) [33]. A hybrid sampling scheme was adopted, which implemented full-area control via a 1 km^2^ grid and prioritized site arrangement by catchment units. The specification requires a sampling density of 4–8 points per square kilometer for 1:50,000 geochemical surveys. The study area features complex lithology and scattered mineralized occurrences; an excessively sparse sampling density may lead to the omission of heavy metal geochemical anomalies. Therefore, rigid regular grid layout was avoided, and the sampling coverage exceeded 75% of the total research area.

Sampling sites were deployed along first- and second-order streams, seasonal gullies and dry valleys, with each catchment controlled within 0.125–0.25 km^2^. Catchment boundaries were delineated using DEM data in a GIS platform. After indoor interpretation, field verification of drainage networks and watershed divides was conducted, following the rule of one sampling site per catchment to prevent cross-watershed sampling. Permanent major rivers are scarce in the study area. Most sampling points lie on the bottoms of gullies with no dry season runoff, where sediments are deposited by floodwater during rainy and snowmelt seasons and comply with the standard definition of stream sediments. A total of 1423 valid samples were obtained in this work.

#### 3.1.2. Field Sampling Procedures

All samples were collected uniformly within a single dry season from May to July 2025. No cross-year sampling or staggered sampling across different hydrological stages was performed to eliminate elemental deviations induced by seasonal hydrological variations. For sampling depth, the stable fine sediment layer at 5–20 cm below the riverbed surface was collected, with the disturbed loose surficial mud of the top 0–5 cm stripped off in advance. Within a 20–30 m upstream–downstream reach of each watercourse, three sediment subsamples were collected and composited into one integrated sample, with coarse gravel, plant residues and artificial construction waste excluded. After air-drying naturally and manual crushing to remove impurities, sediments with particle sizes of 0.25–2 mm were sieved as test materials. All samples were labeled on-site, hermetically packaged and transported to the laboratory for subsequent analysis.

#### 3.1.3. Element Analysis, Instrumentation and Quality Control

Fifteen elements including As, Cd, Cr, Ni, Cu, Pb, Hg, Zn, Mo, Sb, Li, Be, Nb, Ta and Co were determined in this study. The matched analytical techniques adopted are the standard analytical suite for China’s 1:50,000 regional geochemical surveys, and three types of instruments were selected for differentiated testing as follows: (1) Inductively Coupled Plasma Mass Spectrometry (ICP-MS): used for ultra-trace elements such as Cd and Ta, which delivers outstanding detection limits. (2) Inductively Coupled Plasma Optical Emission Spectrometry (ICP-OES): applied to medium- and high-concentration major and minor elements including Cr, Ni and Cu, featuring a wide linear dynamic range without signal saturation at high concentrations. (3) Hydride Generation–Atomic Fluorescence Spectrometry (AFS): designed for hydride-forming elements As, Sb and Hg. Severe spectral interferences occur when these elements are measured using the ICP techniques, while atomic fluorescence spectrometry provides superior sensitivity and stability. The corresponding analytical methods and detection limits for all target elements are listed in Table 1.

The whole analytical process was quality-controlled using national stream sediment certified reference materials (CRMs) of the GBW series, including GBW07301a, GBW07305a, GBW07309 and GBW07312. One set of CRM was inserted every 50 unknown samples and subjected to identical pretreatment and measurement procedures. The recoveries of all target elements ranged from 85% to 115%, the relative error of measured values was ≤10%, and the relative standard deviation (RSD) was less than 5%. These results meet the analytical precision requirements specified in DZ/T 0279-2016 [34] and the standards for 1:50,000 regional geochemical surveys.

### 3.2. Evaluation Methods

The pollution load index (*PLI*) was adopted to calculate the heavy metal pollution grades of stream sediments [35]. The calculation formula is shown as follows:

(1) $$CF_{i}=C_{i}/C_{j}$$

(2) $$PLI=\sqrt[{n}]{CF1\times CF2\times \ldots \times CFn}$$

where *CF_i_* represents the contamination factor of heavy metal *i*; *C_i_* is the measured concentration of heavy metal *i*; and *C_j_* denotes the background concentration of heavy metal *i*. The classification criteria for heavy metal pollution levels are defined as follows: *CF* ≤ 1 indicates no pollution (Grade I); 1 < *CF* ≤ 2 represents slight pollution (Grade II); 2 < *CF* ≤ 3 refers to moderate pollution (Grade III); *CF* ≥ 3 stands for severe pollution (Grade IV). In this study, the pollution classification standards for *PLI* are set as: *PLI* ≤ 1 for no pollution; 1 < *PLI* ≤ 2 for slight pollution; 2 < *PLI* ≤ 3 for moderate pollution; *PLI* > 3 for severe pollution.

The geo-accumulation index method has been widely applied in recent years to assess the pollution of stream sediments by heavy metal elements derived from human activities [36]. The calculation formula is shown as follows:

(3) $$I_{geo}=\log_{2}\left(C_{i}/kB_{i}\right)$$

where *C_i_* is the measured concentration of heavy metal *i*; *B_i_* is the background concentration of heavy metal *i*; *k* is a conversion constant adopted to eliminate variations in background values caused by lithological differences across different regions, with a fixed value of 1.5. In this study, the maximum *I_geo_* value is 4.06, and only a few sampling points have *I_geo_* > 3. Therefore, all samples with *I_geo_* > 2 are uniformly classified as severe pollution in the grading standard; 1 < *I_geo_* ≤ 2 corresponds to moderate pollution; and 0 < *I_geo_* ≤ 1 represents slight pollution; and *I_ge_*_o_ ≤ 0 indicates no pollution.

The potential ecological risks of heavy metals in stream sediments were assessed using the risk index (*RI*) method [37]. The calculation formula is shown as follows:

(4) $$RI=\sum_{j=1}^{n}E_{i}^{j}=\sum_{j=1}^{n}\left(T_{j}\times CF_{j}\right)=\sum_{j=1}^{n}\left(T_{j}\times \frac{C_{i}^{j}}{C_{n}^{j}}\right)$$

where *RI* denotes the potential ecological risk index of heavy metals at sampling site *i*; *E_i_^j^* represents the single potential ecological risk factor of heavy metal *j* at sampling site *i*; *T_j_* is the toxic response factor of heavy metal *j* (THg = 40 > TCd = 30 > TAs = 10 > TCu = TPb = TNi = 5 > TCr = 2 > TZn = 1); *CF_j_* stands for the contamination factor of heavy metal j; *C_i_^j^* is the measured concentration of heavy metal *j* in stream sediments at sampling site *i*; and *C_n_^j^* refers to the background concentration of heavy metal *j*. The ecological hazard grading criteria are as follows: *RI* ≥ 600 indicates very high ecological risk; 300 ≤ *RI* < 600 corresponds to high ecological risk; 150 ≤ *RI* < 300 represents moderate ecological risk; *RI* < 150 means low ecological risk.

## 4. Results

### 4.1. Calculation of Background Values of Heavy Metal Elements in Stream Sediments

Calculations were carried out using datasets from the 1423 field samples in compliance with Specification for Geochemical Reconnaissance (1:50,000) (DZ/T 0011-2015) [33]. Prior to statistical analysis, extreme outliers were screened and removed via the three-sigma rule to guarantee that subsequent statistical parameters reliably represent the regional geochemical background. Afterwards, target elements were grouped by distribution characteristics; the geometric mean was selected for mercury (Hg), which exhibited strong discrete distribution [38]. Arithmetic means were applied to calculate the geochemical background values of conventional elements (Cr, Ni, Cu, Zn, Cd, Pb, As). The final statistical results are presented in Table 2.

### 4.2. Analysis of Heavy Metal Concentration Characteristics in Stream Sediments

As presented in Table 3, statistical analysis based on 1423 surface stream sediment samples yields the average mass fractions of eight target heavy metals: Cr, Ni, Cu, Zn, Cd, Pb, Hg and As, which are 96.95, 20.90, 16.42, 79.70, 0.13, 29.88, 0.0297 and 13.31 mg/kg, respectively. Relative to the regional geochemical background values of the Wandashan area, these mean concentrations are 1.10, 0.63, 0.64, 0.91, 1.61, 1.31, 0.79 and 1.40 times the baseline levels. Specifically, Cr, Cd, Pb and As show evident geochemical enrichment, whereas Ni, Cu, Zn and Hg are depleted relative to background. The coefficients of variation (CV) corresponding to each element are listed as follows: Cr (0.58), Ni (0.93), Cu (0.78), Zn (0.47), Cd (0.58), Pb (0.41), Hg (0.73) and As (1.39). All eight heavy metals possess CV values above 0.4, indicative of strong spatial variability throughout the study domain. Such heterogeneous distribution is jointly governed by two driving factors. Primarily, complex stratigraphic sequences and variable lithologies create inherent natural spatial heterogeneity of element abundances. Secondarily, abnormally elevated metal concentrations observed at discrete sampling sites provide clear evidence that anthropogenic interference has substantially altered the spatial patterns of heavy metals preserved within stream sediments [39].

A total of 15 elements were analyzed in this study. Among them, eight toxic heavy metals (As, Cd, Cr, Ni, Cu, Pb, Hg, Zn) were selected as core indicators for pollution assessment. Li, Be, Nb, Ta, Mo, Co and Sb are stratigraphic and metallogenic indicator elements, which were only used for the analysis of regional geochemical characteristics and excluded from element concentration statistics and the calculation of the pollution load index (*PLI*). The measured stream sediment background values in Table 2 were adopted for *PLI* pollution evaluation, while the local regional background values of the Wandashan area in Table 3 were used to calculate the enrichment coefficient K. These two sets of background values serve different evaluation purposes.

### 4.3. Spatial Distribution Characteristics of Heavy Metals in Stream Sediments

**Figure 2:**
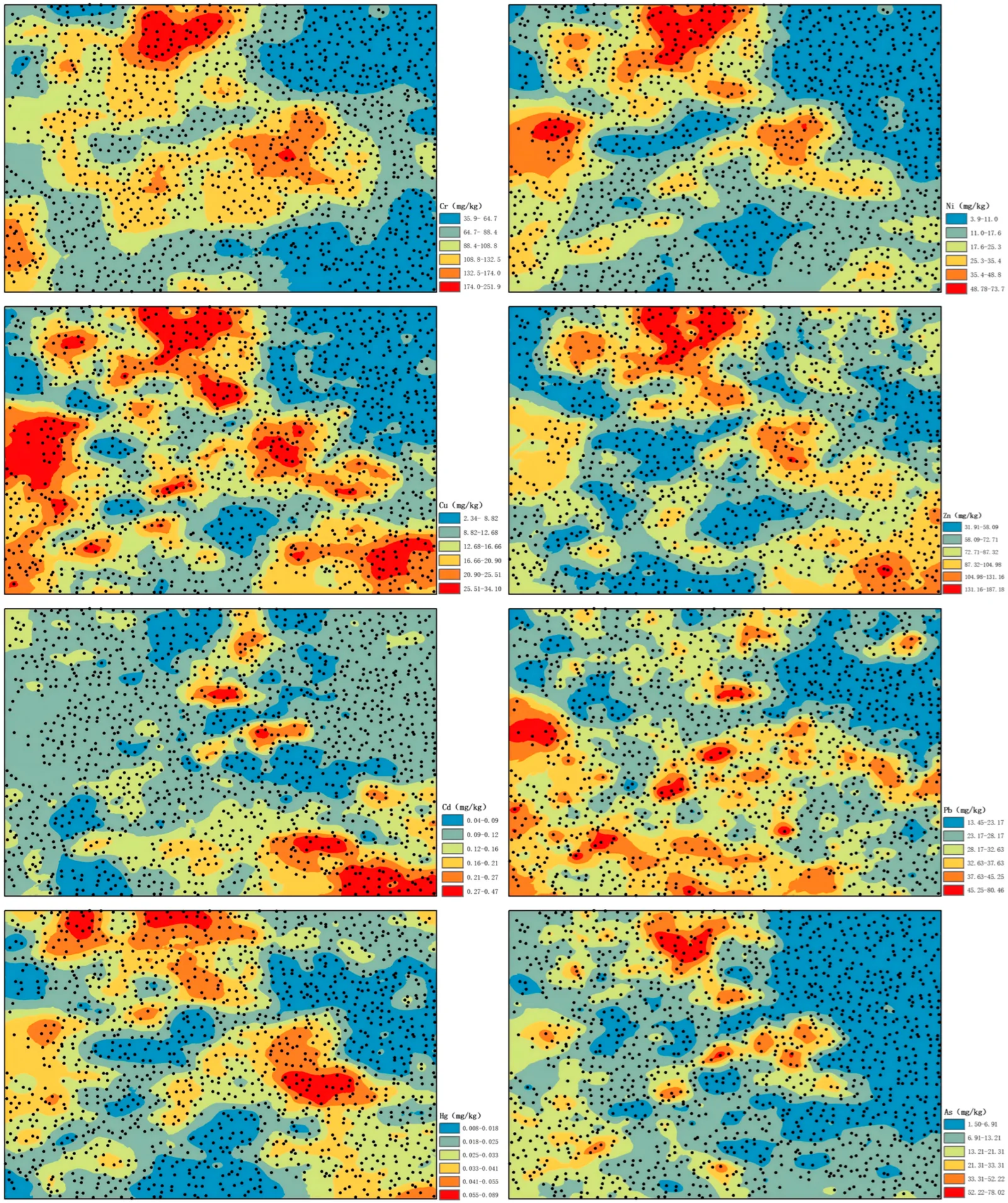
Spatial distribution of heavy metal elements in stream sediments of the study area.

**Figure 3:**
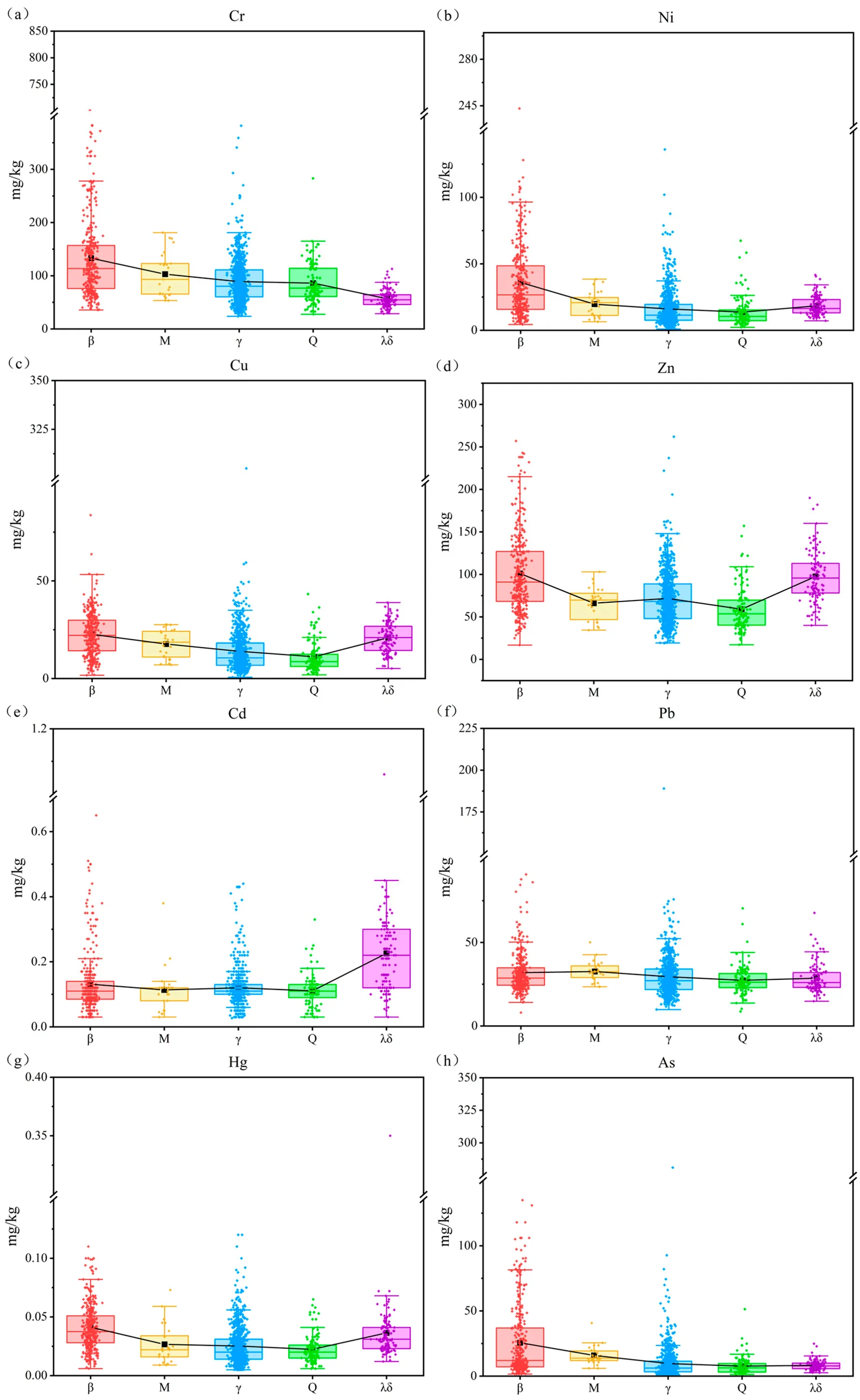
Boxplot of heavy metal distributions in stream sediments of different lithologies (black lines represent the connecting lines of average values; β: basic volcanic rocks; M: metamorphic rocks; γ: granitic intrusive rocks; Q: Quaternary sediments; λδ: intermediate–acid igneous rocks).

We adopted Kriging interpolation to map and interpret the spatial distribution of the eight heavy metals in stream sediments throughout the study area (Figure 2). The interpolation results show that Cr, Ni, Cu and Zn form extensive, continuous high-concentration planar domains, predominantly located in mountainous forested terrain with widespread olivine basalt outcrops in the central study region. Primary ferromagnesian minerals hosted within these basic lithologies undergo chemical weathering, and the resultant mineral detritus is transported and deposited via fluvial processes, ultimately forming the region’s inherent natural high geochemical background for these heavy metals [40]; By contrast, the four elements occur at low concentrations in forested eastern zones dominated by acid granite. To quantitatively clarify lithological constraints on heavy metal enrichment, we performed grouped statistical analysis of eight heavy metal concentrations based on stratigraphic and lithological divisions (Figure 3). The statistical outcomes demonstrate that olivine basalt terrains yield distinctly elevated average contents of Cr, Ni, Cu and Zn, with metamorphic rock areas ranking second. Granitic intrusions and Quaternary sediments feature comparable element levels, whereas intermediate–acid igneous rocks contain the lowest heavy metal concentrations. Collectively, this evidence confirms that lithological variation acts as the primary natural factor controlling the spatial differentiation of Cr, Ni, Cu and Zn throughout the study region [41]. This creates a spatial gradient pattern characterized by enrichment of these metals in central basic rock terrains and depletion in eastern acid rock areas. While the overall distribution of Zn is governed by the geochemical background of basic rocks, distinct zonal enrichment occurs along partial river reaches. Combined with geological data of the study area, iron ore bodies and iron-bearing rock sequences are distributed in the upstream peripheral zones, where iron and zinc minerals commonly occur as associated paragenetic assemblages [42]. Mineral detritus generated by rock weathering migrates and deposits along rivers with surface runoff, resulting in elevated Zn concentrations within river reaches. In contrast, Pb, As and Cd only form patchy local high-concentration hotspots in valley farmland areas, while their contents remain low across extensive pristine forest zones. Long-term application of agrochemicals in valley croplands introduces exogenous heavy metals; meanwhile, fine mineral fragments from peripheral iron and coal mines flow into farmlands via surface runoff, further facilitating localized accumulation of these elements. Previous studies have documented that As, Sb and Pb are typical tracers of agricultural pesticides and fertilizers, and farmland runoff constitutes the primary pathway for exogenous heavy metal input [43]. High-concentration zones of Cd are entirely distributed along the southern Quaternary alluvial–proluvial layers. These Quaternary alluvial–proluvial deposits are predominantly composed of fine clay and silt particles, which feature large specific surface areas and high organic matter contents. Such properties endow the sediments with strong adsorption capacity for heavy metal ions and amplify the enrichment effect of Cd [44]. Hg displays scattered, isolated minor anomalies without contiguous enrichment zones. Its spatial distribution is not governed by exogenous agricultural metal sources, indicating weak anthropogenic interference.

In summary, lithological characteristics constitute the dominant natural factor driving spatial differentiation of heavy metals. The influx of mineral-laden runoff from peripheral mining areas and agrochemical inputs within valley farmlands represent the primary superimposed anthropogenic processes responsible for localized metal enrichment.

### 4.4. Pollution Status and Distribution Characteristics of Heavy Metals in Stream Sediments

The contamination factor (*CF*) values of the eight heavy metals in stream sediments were calculated via the pollution load index method, with the average values ranked in descending order as Hg (1.23) > As (1.18) > Ni (1.08) > Cd (1.06) > Cr (1.05) > Zn (1.029) > Pb (1.028) > Cu (1.018). Hg and As exhibit markedly higher average *CF* values, which identifies them as the predominant pollutant elements in the stream sediments of the study area. When evaluated against standardized pollution grading thresholds, the whole study region falls into the lightly polluted category.

Among the 1423 sampling sites across the study area, Pb has the fewest sites reaching Grade IV contamination factor (*CF*), with only six sites accounting for 0.4% of the total samples. By contrast, As features the largest number of Grade IV sites at 118, which accounts for 8.3% of all samples. For the remaining six heavy metals, the quantity of sites with Grade IV *CF* values ranks in descending order: Ni (80) > Hg (41) > Cd (29) > Cr (25) > Cu (12) > Zn (8). In terms of sites with Grade III *CF* values, the descending order is Hg (148) > Cu (109) > Ni (104) > As (81) > Cd (80) > Cr (52) > Zn (50) > Pb (30). The vast majority of sampling sites fall within Grade I for all eight heavy metals, whose corresponding sample proportions range from 49.5% to 68.5% (Table 4).

The geo-accumulation index (*I_geo_*) method was adopted to calculate the geo-accumulation values and corresponding pollution levels of heavy metals in stream sediments across the study area (Table 5). Among all elements, As contains the largest number of severely polluted sampling sites, totaling 44 and accounting for 3.1% of all samples. The pollution levels of the remaining heavy metals are dominated by slight to moderate pollution. The count of slightly polluted sites follows the descending order: Hg (346) > Cu (289) > Cr (207) = Ni (207) > Zn (198) > Cd (151) = As (151) > Pb (129), with their proportional contributions ranging from 10.6% to 24.3%. For moderately polluted sites, the descending sequence is Ni (77) > As (74) > Hg (37) > Cd (28) > Cr (24) > Cu (11) > Zn (8) > Pb (5), which accounts for 0.4–5.4% of the total samples.

## 5. Discussion

### 5.1. Pollution Grades and Potential Ecological Risks in the Study Area

The pollution load index (*PLI*) results (Table 6) indicate that stream sediments in the study area are dominated by slight pollution. Slightly polluted zones account for 33.2% of the whole region, moderately polluted zones make up 4.1%, and no severely polluted areas are detected. The average *PLI* value of the eight heavy metals across the region is 0.966, corresponding to the unpolluted grade, and contamination is only observed at partial sampling sites. As the predominant contamination factors, Cd and As have average concentrations 1.60 and 1.40 times the stream sediment geochemical background values of the Wandashan area, respectively, representing mild pollution magnitudes. Previous research holds that paragenetic enrichment of Cd and As in surface stream sediments generally arises from the superimposed influences of natural geological background and mining activities [45].

**Figure 4:**
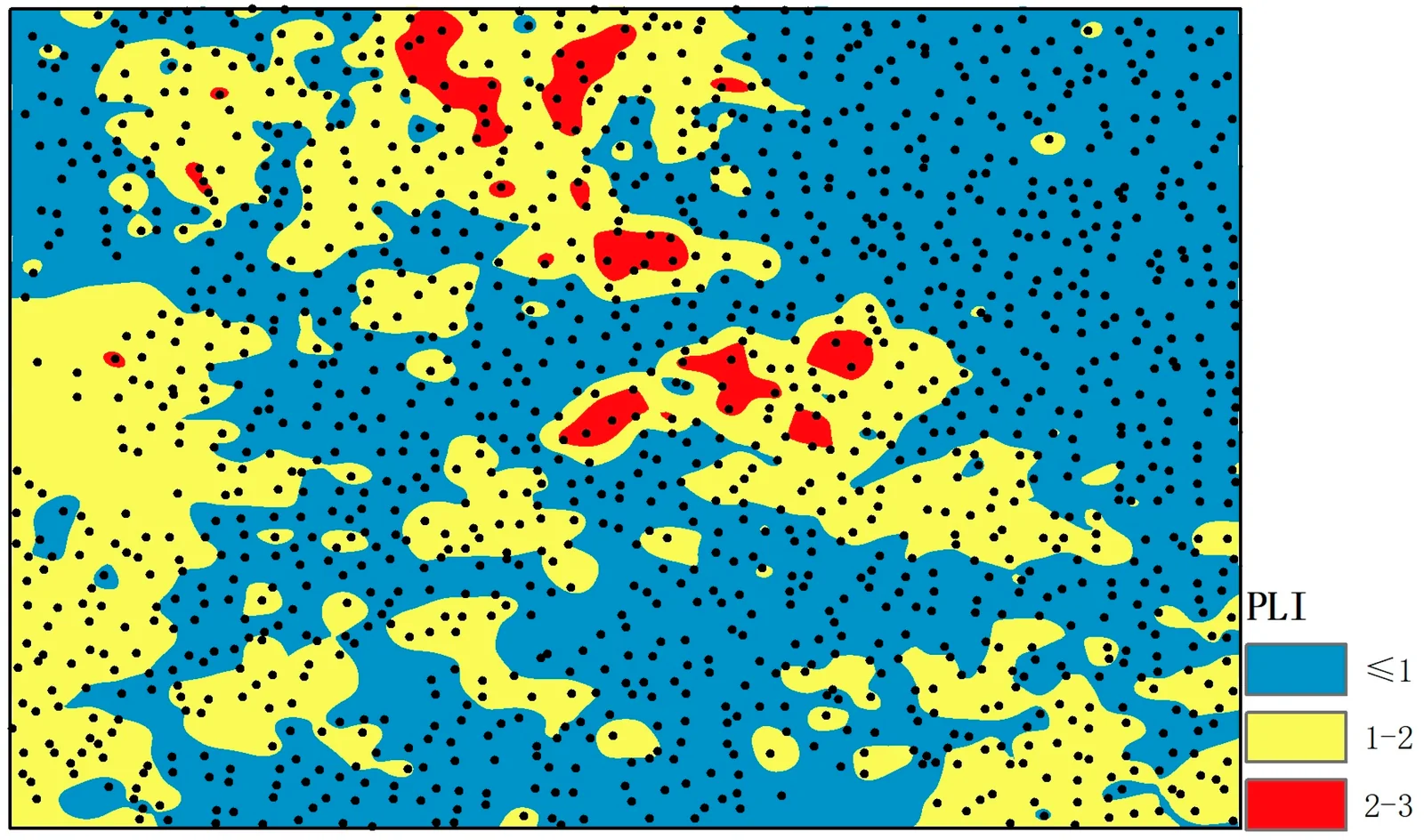
Distribution map of pollution load index (*PLI*) for stream sediments in the study area.

The spatial distribution map of *PLI* values for heavy metals in stream sediments across sampling sites of the study area (Figure 4) reveals that high-*PLI* zones are primarily confined to areas covered by basic rocks. Partial regions here record *PLI* values above 2, corresponding to the moderate pollution grade, while their surrounding areas and valley gullies belong to slight pollution zones with *PLI* values ranging from 1 to 2. By contrast, low-*PLI* zones mainly occur in acid rock outcrop areas, where sediments are generally unpolluted.

**Figure 5:**
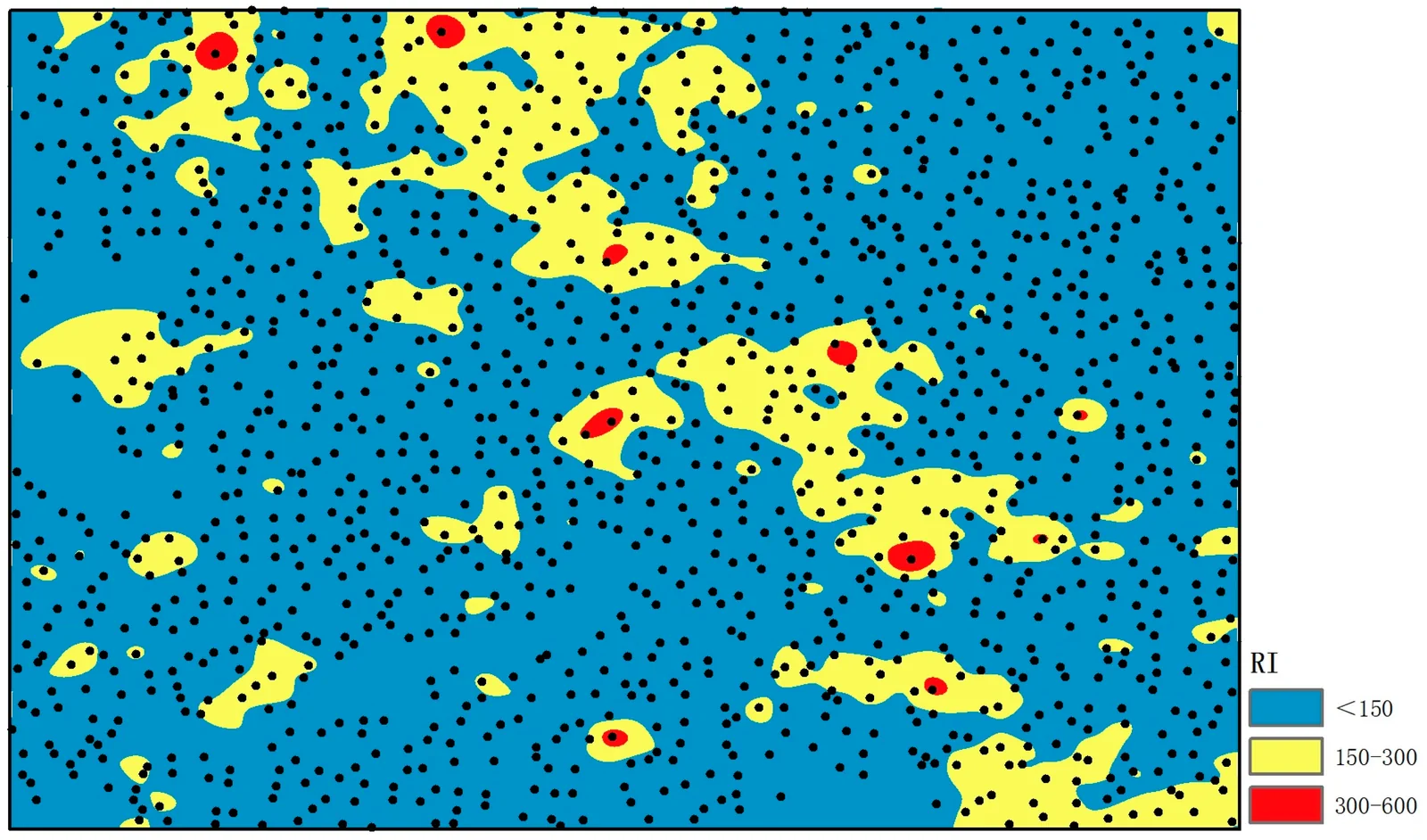
Map of potential ecological risk index (*RI*) levels of heavy metals in stream sediments of the study area.

Analysis of all stream sediment samples from the study area based on the risk index (*RI*) reveals that the average total potential ecological risk index *RI* is 111.45, falling within the slight ecological risk grade. Sites with moderate ecological risk account for 19.2% of all samples, while those with considerable ecological risk make up only 0.8%. The spatial distribution pattern of heavy metal RI values in stream sediments is illustrated in Figure 5. Moderate ecological risk zones are mainly distributed along the olivine basalt–rhyolite contact belt, with isolated individual sampling sites exhibiting considerable ecological risk characteristics.

In this study, four assessment methods were applied separately, namely the contamination factor (*CF*), geo-accumulation index (*I_geo_*), pollution load index (*PLI*), and potential ecological risk index (*RI*). Although the assessment results derived from these four approaches appear divergent, the discrepancy essentially stems from the differing evaluation focuses and research scales of each indicator. Specifically, *CF* and *I_geo_* take individual heavy metals and single sampling sites as evaluation units, focusing on characterizing the anthropogenic accumulation level of a single metal element [46]; *PLI* is calculated comprehensively based on multiple elements and reflects the integrated pollution load at the overall regional scale [47]; In contrast, the *RI* calculation incorporates the toxic coefficients of heavy metals and focuses on assessing potential ecological hazards, and its evaluation results do not fully correspond to the absolute concentrations of elements [48]. In terms of comprehensive assessment outcomes, the study area yields an average *PLI* value of 0.966 and an average *RI* value of 111.45 at the regional scale. Such results demonstrate that the entire region is generally unpolluted with no widespread heavy metal contamination, and slight ecological risk prevails over most terrain. When zooming into individual sampling sites, partial samples register *CF* and *I_geo_* values falling within slight to moderate pollution ranges. These anomalous sites are predominantly concentrated around mining zones and valley agricultural lands, forming scattered spot and patch-shaped anthropogenic pollution hotspots. Taken together, the outputs from the four evaluation methodologies confirm that heavy metal pollution in the study area follows a distinct pattern: the whole watershed remains ecologically safe, while only isolated sampling points exceed pollution thresholds.

### 5.2. Source Identification of Heavy Metal Pollutants in Stream Sediments

#### 5.2.1. Qualitative Source Identification of Heavy Metals in Stream Sediments

**Figure 6:**
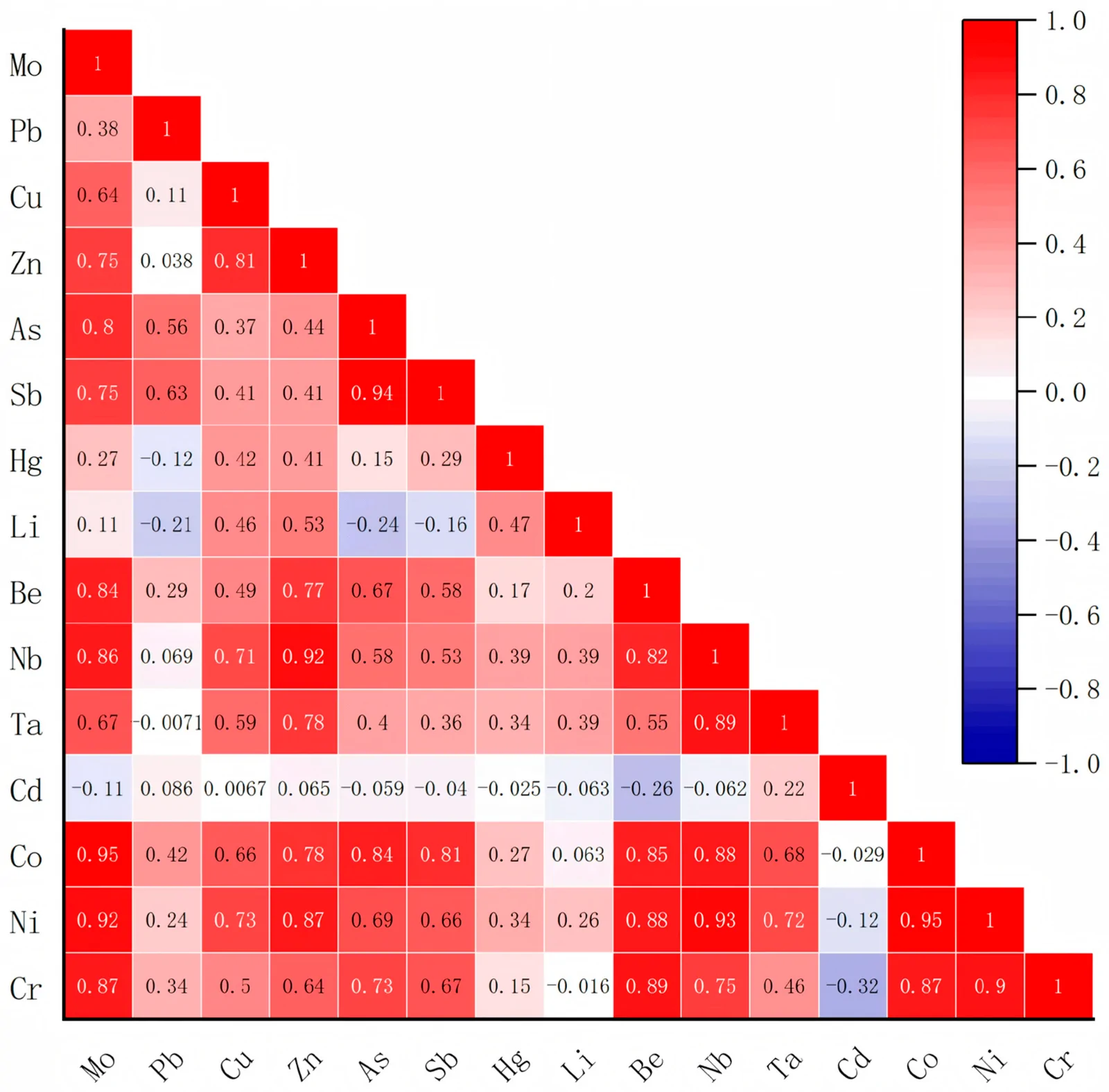
Heatmap of correlation analysis for elements in stream sediments of the study area.

From the perspective of linking macroscopic geochemical behaviors with pollutant origins, Pearson correlation analysis was performed on 15 elements extracted from stream sediment samples of the study area (Figure 6). The analytical results demonstrate strong coupling between elemental paragenetic assemblages and their formative source mechanisms, which can be generalized as a classification of “three element groups with three corresponding material sources” [49]. They indicate contributions derived from late-stage differentiated products of acidic magmas. In contrast, Co, Ni and Cr are compatible elements that reflect material input sourced from ultrabasic and basic rock terranes [50]; Zn tends to migrate jointly with Fe-Mn oxides and sulfide minerals under natural background conditions. Such co-enrichment of elements spanning distinct geochemical categories forms an undisturbed natural geochemical baseline for the study area, free from intense anthropogenic interference. This baseline serves as a vital reference threshold to distinguish anomalous mining-derived anthropogenic pollution signals in later analysis. The second elemental assemblage comprises Cu, Zn, Li, Nb, Ta, Ni and Hg, which display predominantly significant positive correlations with one another (correlation coefficients: 0.32–0.76). Nb, Ta and Li are typical incompatible elements preferentially enriched within felsic lithologies including granite and pegmatite. Their spatial distribution in stream sediments is primarily regulated by parent rock weathering, endowing these elements with remarkable chemical stability and strong spatial inheritance from source bedrocks [51], Ni primarily originates from the weathering of ultrabasic and basic lithologies and frequently co-occurs with Cr, Co and other associated elements within natural sedimentary media. Nevertheless, once these native background elements exhibit statistically significant positive correlations with characteristic mining tracer elements including Cu, Zn and Hg [52]. This demonstrates that mining activities do not develop upon a pristine geochemical blank background, but impose intense superimposed interference on inherent geological substrates. Such coupling reflects a mixed source signature governed by natural geogenic basement and anthropogenic mining disturbances. The third elemental assemblage is composed of Pb, As, Sb and Co. As and Sb display an extremely significant positive correlation (r = 0.80, *p* < 0.01), whereas Pb and Co are significantly positively correlated with both As and Sb (r = 0.44–0.70, *p* < 0.05). Phosphate fertilizers are ubiquitous agrochemical inputs in farming, and their raw phosphate ores commonly host accessory heavy metals including Cd, As and Pb. Continuous long-term fertilization induces gradual enrichment of these metals within topsoil, where they bind with transition metals such as Co to form stable adsorbed complexes [53]. Such elemental co-enrichment acts as a direct signal of anthropogenic non-point agricultural inputs. For the three core elemental assemblages, intra-group correlations are all extremely significant (*p* < 0.01), whereas inter-group correlation coefficients are generally weak (r < 0.4). This stark contrast in correlation significance is strongly coupled with disparate pollutant source types, laying a solid quantitative foundation for source apportionment via subsequent principal component analysis (PCA).

Heavy metal data from stream sediment samples were subjected to normalization. Test results confirmed the dataset’s eligibility for factor analysis (KMO = 0.838, Bartlett’s test of sphericity, *p* < 0.001). On this basis, principal component analysis (PCA) was implemented for all elements in the study area’s stream sediments (Table 7). Three principal components with eigenvalues above 1 were extracted, with eigenvalues of 7.001, 1.687 and 1.343, which together explain 66.886% of the total cumulative variance. The PCA results reveal that PC1 exhibits high factor loadings (>0.614) for Mo, Be, Nb, Co, Ni and Cr. This loading signature is highly consistent with the study area’s geological framework composed of basic rocks, granites and minor metamorphic rocks, thus representing the natural geogenic source. It reflects the integrated geochemical sequence of bedrock weathering, hydraulic transport and sediment enrichment, which fundamentally controls the spatial distribution of heavy metals across the watershed. PC2 is marked by a high-loading elemental suite of Cu, Zn, Hg, Li, Nb, Ta and Ni. This elemental fingerprint matches well with the accessory heavy metal assemblages generated during local iron, gold and coal mining. Coupled with secondary migration of native background elements, PC2 corresponds to a mixed anthropogenic source featuring natural basement overlaid by mining disturbances, which quantifies the extent to which mining activities disrupt regional geochemical cycles. PC3 is dominated by Pb, As, Sb and Co. As and Sb act as characteristic tracers of arsenic- and antimony-containing pesticides; Pb is commonly associated with pesticide auxiliary materials; while Co occurs as an accompanying element from farming practices. Considering the extensive agricultural land around the research zone and sparse traffic flow, PC3 is unambiguously classified as an anthropogenic source dominated by agricultural non-point pollution, which mirrors the secondary adverse effects of agricultural production on fluvial sedimentary environments.

Preliminary outcomes from the preceding correlation analysis reveal cross-group paragenetic associations among Ni, Zn, Nb and Co, which implies these elements are co-governed by a combination of diverse material sources [54]. These four elements also bear noticeable loadings on both PC1 (natural geogenic endmember) and PC2 (mixed mining-related endmember), yielding distinct multi-source loading fingerprints. The relative magnitudes of factor loadings allow differentiation between primary and secondary sources. For Nb and Ni, their stronger loadings on PC1 confirm that natural bedrock weathering constitutes their dominant source, with mining disturbances as a subordinate contributor. By contrast, Zn is governed primarily by mining activities owing to its dominant loading on PC2, where the natural geogenic background only provides baseline elemental concentrations. Co exhibits high loadings not only on PC1 but also on PC3 (agricultural non-point source), following a source pattern dominated by geogenic inputs and supplemented by agricultural inputs. Taken together, the cross-group paragenesis of these elements objectively records the superimposition of diverse geological processes and anthropogenic interference, instead of representing any limitation of the source grouping established via correlation analysis.

#### 5.2.2. Quantitative Source Apportionment of Heavy Metals in Stream Sediments

**Figure 7:**
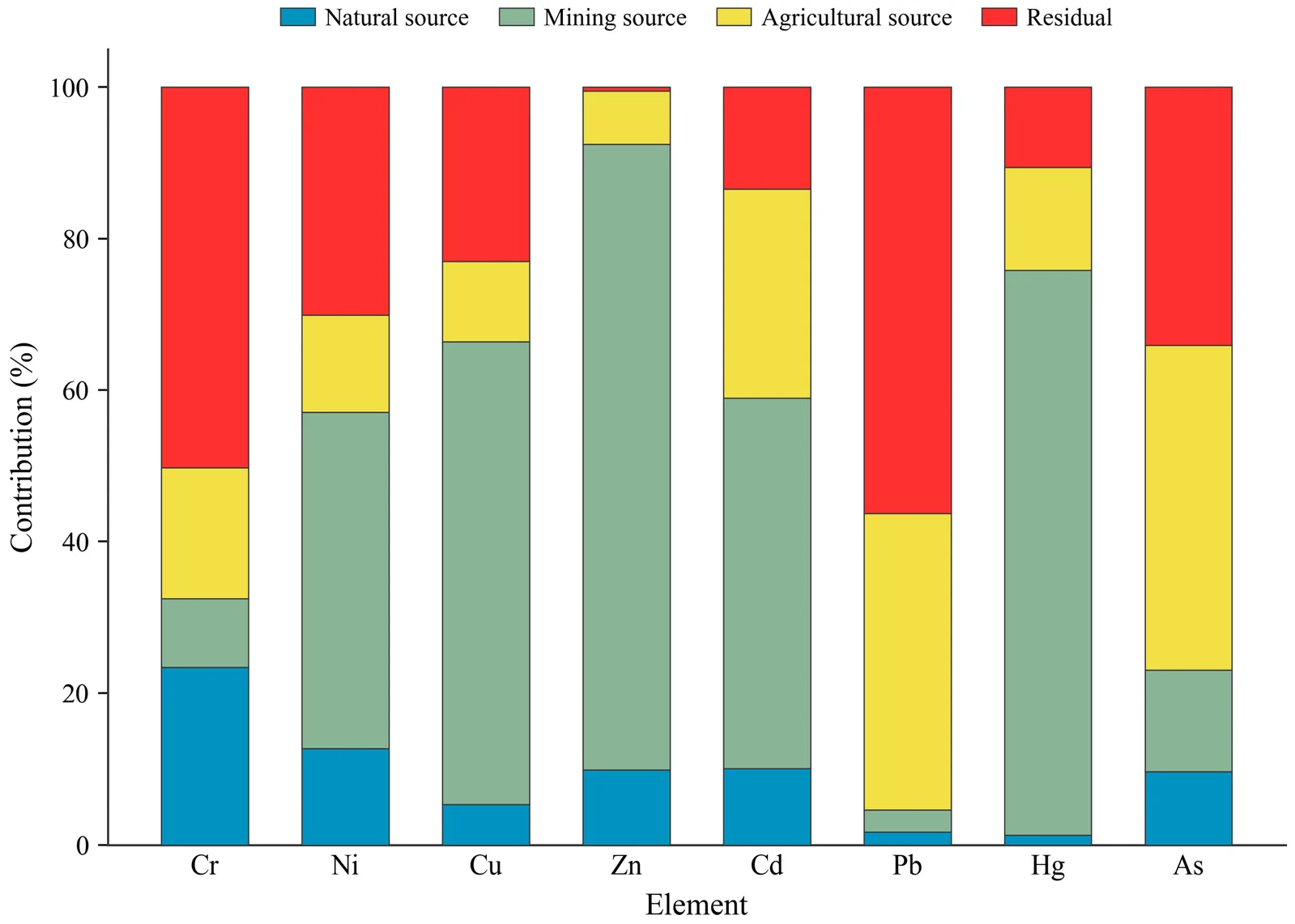
Contribution rates of heavy metal pollution sources in stream sediments of the study area.

An APCS-MLR receptor model was adopted to quantitatively apportion the sources of eight heavy metals (Pb, Cu, Zn, As, Hg, Cd, Ni, Cr) in stream sediments across the study area (Figure 7). The modeling results show considerable variations in the contribution proportions of each source to different heavy metals. The natural geogenic endmember (PC1) presents generally low contribution ratios. It only makes a relatively high contribution to Cr (23.39%), whereas its contributions to all other elements are less than 13%. This proves that geogenic weathering imposes a weak constraint on heavy metal enrichment within the watershed. As the primary anthropogenic endmember, mining activities (PC2) exert dominant control over Zn (82.64%), Hg (74.54%), Cu (61.09%), Cd (48.92%) and Ni (44.42%). This clearly indicates that mineral exploitation and related operations serve as the core driving factor responsible for contamination of the above metals. Agricultural activities (PC3) mainly affect As (42.93%) and Pb (39.16%), which reflects the enrichment effect triggered by agricultural non-point pollution on these two elements. Apart from the three well-defined sources extracted via PCA, a residual source corresponding to unidentified mixed pollution signals also exists in the study area. Four elements receive prominent residual contributions: Pb (56.29%), As (34.06%), Cr (50.26%) and Ni (30.11%). Combined with the anthropogenic activity patterns of the study region, this residual fraction is tentatively attributed to the following pathway: ① Dry and wet atmospheric deposition of heavy metals released from local industrial and mining facilities [55]; ② dispersed pollution sources including road dust [56]; ③ exogenous fluvial runoff transported from upstream catchments [57]. Moreover, the residual source accounts for merely 0.50% of Zn’s total contribution, which further corroborates that the enrichment of Zn is overwhelmingly governed by mining disturbances. Synthesizing all APCS-MLR modeling outputs, mining operations represent the predominant anthropogenic source triggering heavy metal contamination within the watershed; agricultural activities exert a marked influence on the accumulation of As and Pb, whereas contributions derived from natural geogenic basement stay marginal across all metals.

As demonstrated in the foregoing spatial pattern analysis, the macroscopic spatial distributions of Cr, Ni, Cu and Zn are rigidly regulated by regional lithological conditions. Element concentrations are generally high within basic rock outcrops yet relatively low across felsic rock terrains, which confirms that the natural geogenic background dominates the baseline heavy metal contents. However, APCS-MLR modeling outputs indicate that the contribution rates of geogenic sources to these elements are mostly less than 23%. While this contrast appears contradictory at first glance, it stems from the divergent analytical dimensions adopted by the two research methods. The PCA-APCS-MLR receptor modeling framework is designed to quantify anomalous heavy metal surpluses above the regional natural geochemical baseline, with its core objective to calculate the proportion of heavy metals introduced via anthropogenic activities. The whole study watershed hosts a relatively stable inherent geochemical substrate; baseline element loads derived from bedrock weathering are not categorized as anomalous surpluses in model operation, which accounts for the low contribution proportions assigned to the geogenic endmember. To summarize, regional lithology shapes the overall spatial variation and native background concentrations of heavy metals, whereas anthropogenic disturbances such as mining and agricultural practices serve as the prime drivers responsible for localized extreme enrichment and point-source pollution. The two sets of results interpret two independent layers of information: large-scale geogenic baseline features and small-scale anthropogenic excess increments, and thus no logical inconsistency exists between them.

In aggregate, the findings derived from correlation analysis, principal component analysis and the APCS-MLR receptor model cross-validate each other. Together, they uncover a three-endmember source regime governing heavy metal distributions within the watershed: natural geogenic basement constitutes the foundational background, mining disturbances represent the predominant anthropogenic input, and agricultural non-point sources act as a supplementary contributor. This integrated evidence lays a robust scientific foundation for heavy metal source tracing and precise pollution management across the study region.

## 6. Conclusions

(1) This research carried out pollution assessment and quantitative source apportionment of heavy metals in stream sediments from the hilly zone at the western foot of the Wanda Mountains. The analytical results reveal evident enrichment of Cr, Cd, Pb and As, alongside depletion of Ni, Cu, Zn and Hg. All target heavy metals display strong spatial variability, which is jointly controlled by stratigraphic lithology and diverse anthropogenic disturbances. In terms of spatial patterns, the concentrations of Cr, Ni, Cu and Zn are tightly constrained by local lithological conditions: higher values prevail over basic rock outcrops, whereas lower concentrations occur in felsic rock terrains. Local anomalous accumulation of Zn correlates closely with iron mining activities in upstream catchments. High contents of Pb, As and Cd within agricultural zones stem from long-term pesticide application and input of terrigenous mineral detritus. By comparison, Hg receives minimal anthropogenic interference across the study watershed.

(2) Pollution assessment demonstrates that the watershed generally suffers mild contamination, with an average pollution load index (*PLI*) of 0.966 indicating an unpolluted background, despite contaminated individual sampling sites. Hg and As act as the dominant pollutant elements; the mean concentrations of Cd and As are 1.60 and 1.40 times their corresponding geochemical background values, respectively. Calculations of the geo-accumulation index (*I_geo_*) show that all elements only pose slight to moderate pollution levels, apart from As, at which several severely polluted sites were identified. The potential ecological risk index (*RI*) averages 111.45, corresponding to low overall ecological hazard, whereas moderate ecological risk zones occur within the olivine basalt–rhyolite lithological belt. Taken together, the heavy metal pollution pattern of the study region can be generalized as “generally safe across the whole watershed with localized sampling sites exceeding threshold limits”. Such anomalous points are primarily concentrated around mining districts and river valley farmlands, consistent with the spatial distribution characteristics elaborated above.

(3) Source apportionment identifies a distinct three-assemblage three-endmember source pattern consistent with the foregoing correlation and PCA results. The natural geogenic endmember, featuring high loadings of Mo, Be, Nb, Co, Ni, Cr and Zn, stems predominantly from regional parent rock weathering. The mixed mining-associated endmember with Cu, Zn, Hg, Li, Nb, Ta and Ni closely corresponds to local mineral extraction activities. The agricultural non-point endmember dominated by Pb, As, Sb and Co records the geochemical signals induced by farming practices. Quantitative APCS-MLR calculations further confirm that mining disturbances constitute the primary anthropogenic contributor to Zn, Hg, Cu, Cd and Ni; agricultural activities exert prominent control over the enrichment of As and Pb, while contributions from natural geogenic basement remain trivial. Beyond the three identified endmembers, the residual source is tentatively attributed to dry–wet atmospheric deposition, road dust emissions and exogenous fluvial runoff transported from upstream watersheds. This study underscores that regional lithology sets the baseline concentration levels of heavy metals, whereas anthropogenic interferences, including mining and agricultural production, function as the key drivers triggering localized anomalous enrichment and point pollution.

## Figures and Tables

**Table 1:** Analysis methods and detection limits of indicators for stream sediment samples.

Indicator	Analytical Methods	Detection Limits	Indicator	Analytical Methods	Detection Limits
As	Hydride Generation–Atomic Fluorescence Spectrometry	0.5 mg/kg	Mo	Inductively Coupled Plasma Optical Emission Spectrometry	0.20 mg/kg
Cd	Inductively Coupled Plasma Mass Spectrometry	0.044 mg/kg	Sb	Hydride Generation–Atomic Fluorescence Spectrometry	0.05 mg/kg
Cr	Inductively Coupled Plasma Optical Emission Spectrometry	4.92 mg/kg	Li	Inductively Coupled Plasma Optical Emission Spectrometry	1.0 mg/kg
Ni	Inductively Coupled Plasma Optical Emission Spectrometry	0.93 mg/kg	Be	Inductively Coupled Plasma Optical Emission Spectrometry	0.05 mg/kg
Cu	Inductively Coupled Plasma Optical Emission Spectrometry	0.2 mg/kg	Nb	Inductively Coupled Plasma Optical Emission Spectrometry	0.01 mg/kg
Pb	Inductively Coupled Plasma Optical Emission Spectrometry	0.1 mg/kg	Ta	Sodium Peroxide Fusion–Inductively Coupled Plasma Mass Spectrometry	0.1 mg/kg
Hg	Vapor Generation–Cold Vapor Atomic Fluorescence Spectrometry	0.005 mg/kg	Co	Inductively Coupled Plasma Optical Emission Spectrometry	0.20 mg/kg
Zn	Inductively Coupled Plasma Optical Emission Spectrometry	2.0 mg/kg			

**Table 2:** Calculation results of background values for heavy metal elements in stream sediments (mg/kg).

Indicator	Cr	Ni	Cu	Zn	Cd	Pb	Hg	As
Background value	92.3	19.3	16.1	77.5	0.12	29.1	0.024	11.2

**Table 3:** Statistical characteristics of heavy metal element contents in stream sediments of the study area.

Item	Cr	Ni	Cu	Zn	Cd	Pb	Hg	As
Maximum (mg/kg)	701.0	243.0	305.0	262.0	1.06	189.0	0.35	281.0
Minimum (mg/kg)	23.7	0.9	0.6	16.8	0.027	8.0	0.005	1.1
Mean (mg/kg)	96.9	20.9	16.4	79.7	0.131	29.9	0.029	13.31
Standard deviation (mg/kg)	56.7	19.5	12.8	37.4	0.076	12.2	0.022	18.5
Coefficient of variation/%	58.5	93.2	78.1	47.0	58.4	40.8	72.8	139.4
Geochemical background value of Wandashan area (mg/kg)	88.3	33.1	25.6	87.3	0.081	22.7	0.043	9.5
K value	1.10	0.63	0.64	0.91	1.61	1.31	0.70	1.40

Note: The K value is the ratio of the average content to the background value of the Wandashan area.

**Table 4:** Number of sampling sites at different CF pollution levels in the study area.

Evaluation Index		No Pollution	Light Pollution	Moderate Pollution	Severe Pollution
*CF*	Cr	816	530	52	25
	Ni	915	324	104	80
	Cu	814	488	109	12
	Zn	786	579	50	8
	Cd	975	339	80	29
	Pb	823	564	30	6
	Hg	704	530	148	41
	As	970	254	81	118

**Table 5:** Geo-accumulation index of heavy metals in stream sediments of the study area (data in parentheses: the proportion of sampling sites to the total number of samples; other data: the number of sampling sites).

Geo-Accumulation Index	*I_geo_* ≤ 0	0 < *I_geo_* ≤ 1	1 < *I_geo_* ≤ 2	2 < *I_geo_* ≤ 3
Pollution Level	No Pollution	Light Pollution	Moderate Pollution	Severe Pollution
Cr	1191 (83.7)	207 (14.5)	24 (1.7)	1 (0.1)
Ni	1136 (79.8)	207 (14.5)	77 (5.4)	3 (0.2)
Cu	1122 (78.8)	289 (20.3)	11 (0.8)	1 (0.1)
Zn	1217 (85.5)	198 (13.9)	8 (0.6)	0 (0.0)
Cd	1243 (87.4)	151 (10.6)	28 (2.0)	1 (0.1)
Pb	1288 (90.5)	129 (9.1)	5 (0.4)	1 (0.1)
Hg	1036 (72.8)	346 (24.3)	37 (2.6)	4 (0.3)
As	1154 (81.1)	151 (10.6)	74 (5.2)	44 (3.1)

**Table 6:** Proportion of sampling sites at various *PLI* pollution levels in the study area/%.

Evaluation Index	No Pollution	No Pollution	Moderate Pollution	Severe Pollution
*PLI*	62.8	33.2	4.10	0.00

**Table 7:** Principal Component Analysis of Elements in Stream Sediments of the Study Area.

Indicator	PC1	PC2	PC3
Mo	0.618	0.359	0.422
Pb	0.086	0.030	0.744
Cu	0.259	0.616	0.195
Zn	0.434	0.748	0.116
As	0.447	0.128	0.749
Sb	0.334	0.161	0.783
Hg	0.024	0.555	0.177
Li	0.071	0.735	−0.118
Be	0.754	0.246	0.148
Nb	0.614	0.676	0.130
Ta	0.243	0.697	0.178
Cd	−0.440	0.443	0.459
Co	0.641	0.400	0.531
Ni	0.706	0.510	0.269
Cr	0.830	0.066	0.229
Eigenvalue	7.001	1.689	1.343
Variance/%	46.670	11.260	8.955

Rotation Method: Varimax with Kaiser normalization; rotation converged after 3 iterations.

## Data Availability

The data presented in this study are available on request from the corresponding author.
